# Antioxidant Compounds from Propolis Collected in Anhui, China

**DOI:** 10.3390/molecules16043444

**Published:** 2011-04-21

**Authors:** Haisha Yang, Yuqiong Dong, Huijing Du, Haiming Shi, Yunhua Peng, Xiaobo Li

**Affiliations:** 1 School of Pharmacy, Shanghai Jiao Tong University, No. 800 Dongchuan Road, Minhang District, Shanghai 200240, China; E-Mails: YHS@sjtu.edu.cn (H.Y.); dyq1015@126.com (Y.D.); du1212du@163.com (H.D.); 2 Shanghai Key Laboratory for Compound Chinese Medicines, Institute of Chinese Materia Medica, Shanghai University of Traditional Chinese Medicine, 1200 Cailun Road, Zhangjiang Hi-Tech Park, Shanghai 201210, China; 3 School of Agriculture and Biology, Shanghai Jiao Tong University, No. 800 Dongchuan Road, Minhang District, Shanghai 200240, China; E-Mail: yhpeng@sjtu.edu.cn (Y.P.)

**Keywords:** propolis, antioxidant activity, phenolics, natural antioxidants

## Abstract

The antioxidant activities of the chloroform, ethyl acetate and *n*-butanol extract fractions from propolis collected in Anhui, China were evaluated in this study. The ethyl acetate fraction contained the highest amount of total phenolics and total flavonoids, and showed the greatest 1,1-diphenyl-2-picrylhydrazyl radical (DPPH) and 2,2′-azino-bis(3-ethylbenzothiazoline-6-sulfonic acid) diammonium salt (ABTS) radical scavenging capacities and Ferric Reducing/Antioxidant Power (FRAP). The antioxidant activity of twenty-two compounds isolated from the ethyl acetate fraction was also evaluated using the above-mentioned three assays. The results indicated that phenolics contributed to the antioxidant activity of propolis collected in Anhui, China. Therefore, propolis collected in Anhui, China and its phenolics might be used as a natural antioxidant.

## 1. Introduction

It is well-known that oxidation damages various biological substances and subsequently causes many diseases, such as carcinogenesis, diabetes, or atherosclerosis [1,2,3]. Consequently, antioxidants play an important role in reducing the risk of a wide spectrum of chronic diseases. Recently, identification and isolation of new antioxidants from natural sources has become an active area of research, as a number of natural products, such as flavonoids, phenolics or terpenes, isolated from plants and foods have shown potent antioxidant activity [4,5,6].

Propolis is a resinous hive product collected by honeybees from various plant sources. The use of propolis goes back to ancient times, at least to 300 BC, and it has been used as a medicine in local and popular medicine in many parts of the world, both internally and externally. In recent years, propolis has attracted researchers’ interest because of its many beneficial biological effects, such as hepatoprotective, antitumour, antioxidative, antimicrobial, anti-inflammatory activity [7,8]. Besides, propolis-containing products have been intensely marketed by the pharmaceutical industry and health-food stores. 

The chemical composition of propolis depends on the specificity of the local flora at the site of collection. More than 300 constituents have been identified from propolis, among which phenolic compounds such as flavonoids, phenolic acids and phenolic acid esters have been reported as major constituents of propolis from the temperate zone [8]. In our continuing search for potent antioxidants from natural sources [9,10], we found that the ethanolic extract of propolis collected in Anhui, China displayed excellent antioxidant effects in the DPPH, ABTS free radical scavenging and ferric reducing assays. Thus, we evaluated the antioxidant activities of the ethanolic extract from propolis collected in Anhui, China and its fractions and isolated compounds with potent antioxidant activities by bio-isolation.

## 2. Results and Discussion

### 2.1. Structure Elucidation of Isolated Compounds 

**Figure 1:**
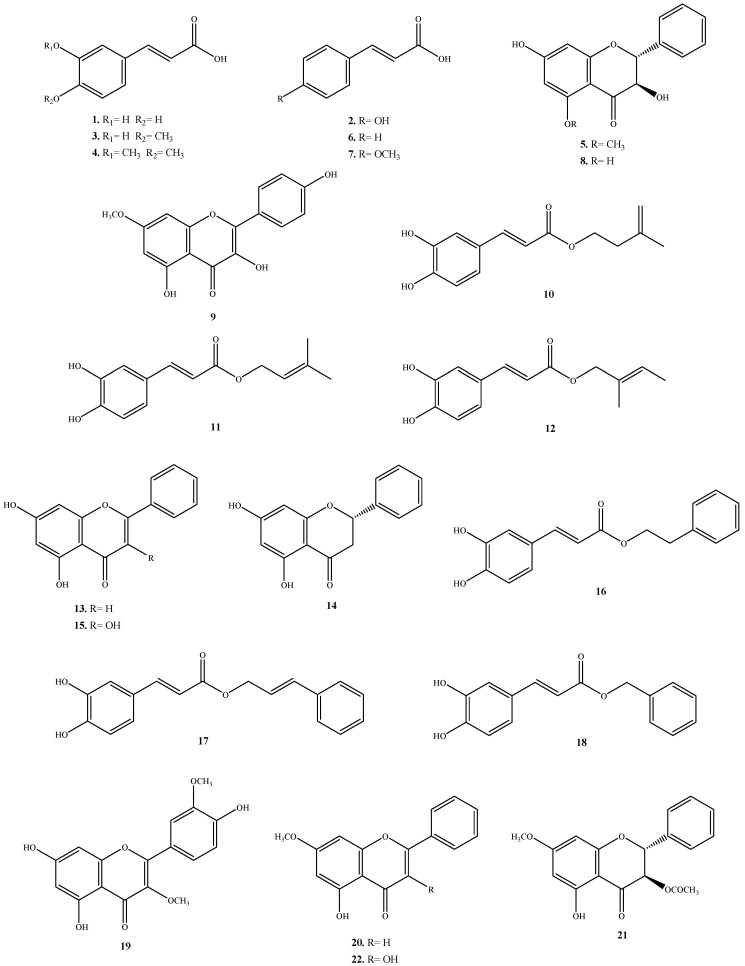
Structures of compounds **1**-**22** from propolis collected in Anhui, China.

The chemical structures of twenty-two isolated compounds: caffeic acid (**1**), (*E*)-*p*-coumaric acid (**2**), isoferulic acid (**3**), 3,4-dimethylcaffeic acid (**4**), pinobanksin-5-methyl ether (**5**) [11], cinnamic acid (**6**), 4-methoxycinnamic acid (**7**), pinobanksin (**8**) [12], rhamnocitrin (**9**) [13], isopent-3-enyl caffeate (**10**) [14], 3,3-dimethylallyl caffeate (**11**) [14], 2-methyl-2-butenyl caffeate (**12**) [15], chrysin (**13**) [12], pinocembrin (**14**) [12], galangin (**15**) [16], phenethyl caffeate (**16**) [17], cinnamyl caffeate (**17**) [18], benzyl caffeate (**18**) [19], quercetin-3,3’-dimethyl ether (**19**) [20], tectochrysin (**20**) [21], 3-acetylpinobanksin-7-methyl ether (**21**) [22] and galangin-7-methyl ether (**22**) [23] were confirmed by comparing HPLC, high performance thin layer chromatography (HPTLC) and characteristic spectroscopic data (UV, IR, MS and NMR) with that of standards or with values reported in the literature. The chemical structures of the isolated compounds are shown in Figure 1. This is the first report of compounds **9**, **10**, **12** and **21** from propolis collected in China.

### 2.2. Total Phenolic Content, Total Flavonoids Content and Antioxidant Activities of Ethanolic Extract and its Three Fractions

The contents of phenolic compounds in the ethanolic extract of propolis collected in Anhui, China and its three fractions varied from 174.7 to 235.6 μg gallic acid mg^-1^ dry sample (Table 1), and the EA fraction was found to contain the most of phenolic compounds. Although phenolic-type compounds have been attributed to the antioxidant activities of plants and the major determinant of antioxidant potentials of foods [24], good antioxidant activities do not always correlate with the presence of large quantities of phenolic-type compounds.

Total flavonoids contents, which is believed to be another determinant of the overall antioxidant activities, were measured and the results are displayed in Table 1. The quantities of flavanoids in the ethanolic extract and three fractions of propolis collected in Anhui, China were found to vary from 34.5 μg to 62.1 μg quercetin mg^-1^ dry sample. As in the above-mentioned phenolic case, the EA fraction also contained the most flavanoids. The quantities of flavanoids were found to decrease in the following order: EA > CF > EE > BT.

**Figure 2:**
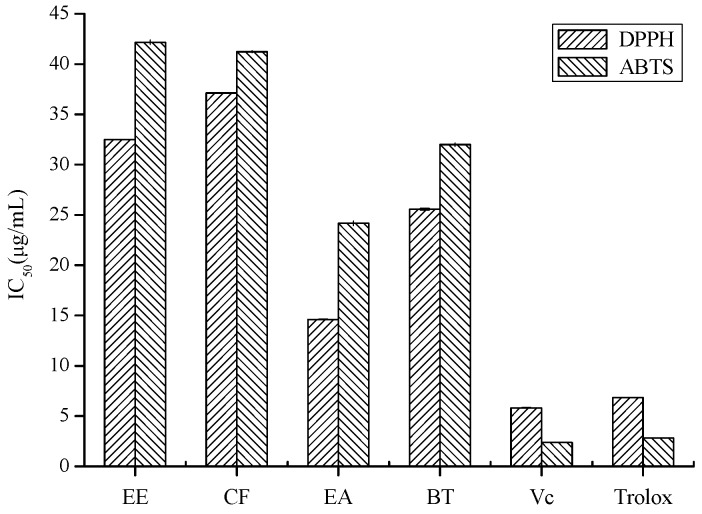
The DPPH and ABTS radical scavenging acitivities of ethanolic extract and three fractions from propolis collected in Anhui, China (EE, CF, EA, BT represent the ethanolic extract, chloroform, ethyl acetate and *n*-butanol fractions, respectively; L-Ascorbic acid (Vc) and Trolox were used as positive controls.

**Figure 3:**
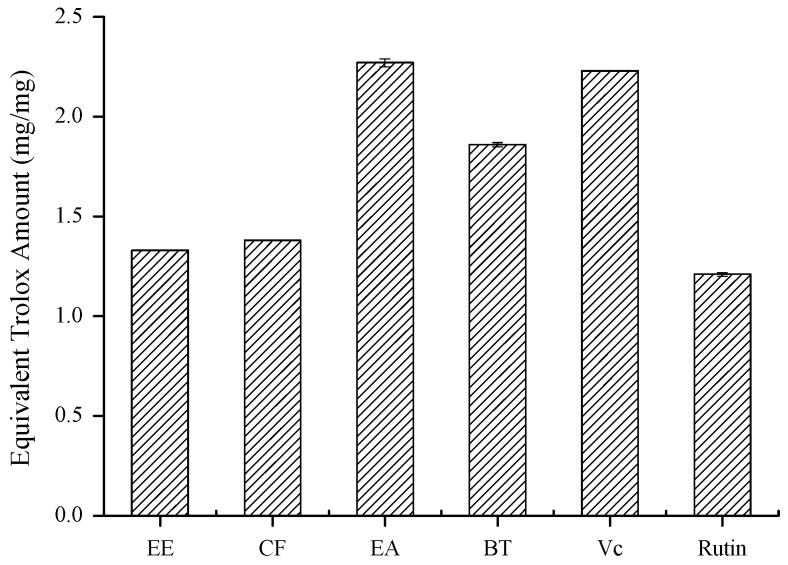
The reducing power of ethanolic extract and three fractions from propolis collected in Anhui, China (EE, CF, EA, BT represent the ethanolic extract, chloroform, ethyl acetate and *n*-butanol fractions, respectively; Vc and rutin were used as positive controls).

Oxidation is a very complex process with different mechanisms. Thus, there is no single method which correctly evaluates the total antioxidant capacity. The method should be selected considering the function to be evaluated, so we used two different radical scavenging assays and one reductive potential assay to measure antioxidant activity of samples. The EA fraction had the strongest DPPH and ABTS radicals scavenging abilities (Figure 2), and possessed significant reducing power over other fractions (Figure 3), indicating that the antioxidant of propolis collected in Anhui, China ethanolic extract had been efficiently enriched in the EA fraction. To trace the bioactive compounds, the EA fraction was further fractionated by using an array of chromatographic techniques.

### 2.3. DPPH, ABTS Scavenging Activity Test of the Compounds Isolated from Chinese Propolis.

The DPPH and ABTS radical scavenging assays were used to evaluate the antioxidant activities of all isolated compounds. A phenolic acid (compound **1**), one flavonoid (compound **15**) and three phenolic acid esters (compounds **16**-**18**) exhibited excellent DPPH radical scavenging activities (Table 2). Compound **1** had the highest antioxidant activity and indeed this was stronger than positive controls (Vc, Trolox and rutin). Compounds **15**-**18** were also highly active, with IC_50_ values from 5.87 to 17.02 μg/mL. Three phenolic acids (compound **1-3**), one flavonoid (compound **15**) and three phenolic acid esters (compound **16**-**18**) showed potent ABTS radical scavenging activities (Table 2). Compound **1** also possessed the strongest antioxidant activity. Among the flavanoids obtained, only compound **15** showed stronger activity in two assays, indicating that the number and position of hydroxyls present in the aromatic ring were crucial to maintain antioxidant activity. In addition, it is noteworthy that isolated phenolic acid and flavanoids with a methoxyl group in the aromatic ring exhibited no free radical scavenging capacity. A possible explanation for this result is the difficulty of quinone formation involved in the reaction mechanism between phenolics and free radicals [25].

### 2.4. Ferric Reducing/Antioxidant Power (FRAP) Assay

The FRAP values expressed as Trolox equivalents (μg Trolox μg^-1^ sample dry weight), were found to vary from 1.39 to 5.82 μg. The strongest antioxidant, based on the FRAP assay, was compound **16**, consistent with its stronger abilities in the DPPH and ABTS radical assays. The FRAP assay quantifies chemical reductants that would reduce the ferric complex to the ferrous form and it is known that not all of these reductants are antioxidants [26]. It is also true in our study where compound **15** exhibited the highest activity scavenging of DPPH and ABTS radicals, but did not show consistent reducing power. In view of our results (Table 2), it appears that phenolics having *ortho*-dihydroxyl groups showed stronger activities.

## 3. Experimental 

### 3.1. Chemicals and Reagents

Reference compounds [caffeic acid, (*E*)-*p*-coumaric acid, isoferulic acid, 3,4-dimethylcaffeic acid, cinnamic acid and 4-methoxycinnamic acid] were purchased from Aladdin Reagent Company (Shanghai, China). Folin-Ciocalteu phenol reagent was obtained from Applichem Co. (Darmstadt, Germany). 1,1-Diphenyl-2-picrylhydrazyl radical (DPPH), L-ascorbic acid (Vc), 2,2′-azino-bis(3-ethylbenzothiazoline-6-sulfonic acid) diammonium salt (ABTS), 1,3,5-tri(2-pyridyl)-2,4,6-triazine (TPTZ), 6-hydroxy-2,5,7,8-tetramethylchromancarboxylic acid (Trolox) were purchased from Sigma-Aldrich Chemical Co. (St. Louis, MO, USA). Deionized water was obtained with a Milli-Q Water purification system (Millipore, MA, USA). All other chemicals and reagents were of analytical grade.

### 3.2. Plant Material

Crude propolis were collected in August 2008, from Anhui Province, China, and authenticated by Mengyue Wang. Voucher specimen (FJ080812) has been deposited at the herbarium of the School of Pharmacy, Shanghai Jiao Tong University, Shanghai, China.

### 3.3. Apparatus

The optical absorbance was measured in an xMark Microplate Absorbance Spectrophotometer (Bio-Rad Co. Ltd., USA) with a spectral range from 200 to 1000 nm and 1 nm step size. ^1^H-NMR and ^13^C-NMR spectra were recorded on Bruker Avance III 400 and Bruker Avance DRX-500 spectrometers. ESI-MS analyses were recorded on an Agilent 6410 triple-quad mass spectrometer. Semipreparative RP-HPLC was performed on a Shimadzu LC 2010A liquid chromatography system, using a Waters nova-pak C_18_ column (7.8 × 300 mm, 6 μm, 60 Å) (Milford, MA, USA). The column temperature was set at 35 °C. UV absorption was monitored at 210 nm.

### 3.4. Preparation of Extract and Fractions

Crude propolis (2 kg) were extracted three times with 95% ethanol (10 L) in an ultrasonic water bath for 1.5 h each time. The combined ethanolic extract was completely evaporated under reduced pressure to afford a brownish red residue (650 g). The resulting ethanolic extract (EE) was suspended in water, then partitioned in ascending polarity manner with chloroform, ethyl acetate and *n*-butanol to yield the corresponding soluble fractions. The yields of chloroform fraction (CF), ethyl acetate fraction (EA) and *n*-butanol fraction (BT) were 400 g, 72 g and 55 g, respectively. The ethyl acetate fraction which showed the best antioxidant activity was taken for further fractionation and isolation of individual compounds.

### 3.5. Isolation and Identification of Antioxidant Compounds

The ethyl acetate fraction (72 g) was chromatographed directly on silica gel and eluted with a gradient system of methanol and chloroform to afford ten fractions (Frs.1-10), which were further separated to yield individual compounds. Specifically, Fr.2 was chromatographed on Sephadex LH-20 (CHCl_3_: MeOH = 1: 1) and further purified by HPLC (MeOH: H_2_O = 65: 35) to afford compounds **20** (8 mg), **21** (6 mg) and **22** (9 mg), respectively. Fr.3 was subjected to column chromatography over Sephadex LH-20 (MeOH) and further purified by HPLC (MeOH: H_2_O = 55: 45) to yield compounds **17** (3 mg), **18** (2.5 mg) and **19** (4 mg), respectively. Compound **16** (75 mg) was isolated from Fr.4 by a combination of silica gel (petroleum ether: acetone = 15: 1) and Sephadex LH-20 (MeOH) column chromatography. Fr.5 was chromatographed on Sephadex LH-20 column chromatography (MeOH) and further purified by HPLC (MeOH: H_2_O = 45: 55) to afford compounds **10** (1.5 mg), **11** (1.0 mg), **12** (1.2 mg), **13** (86 mg), **14** (75 mg) and **15** (56 mg), respectively. Compounds **8** (15 mg) and **9** (7 mg) were obtained from Fr.6 by Sephadex LH-20 (MeOH) column chromatography. Fr.7 was chromatographed on Sephadex LH-20 column chromatography (MeOH) and further purified by HPLC (MeOH: H_2_O = 40: 60) to yield compounds **5** (28 mg), **6** (10 mg) and **7** (8 mg), respectively. Fr.8 and Fr.9 were subjected to column chromatography over Sephadex LH-20 (MeOH: H_2_O = 1: 1) and further purified by HPLC (MeOH: H_2_O = 30: 70) to yield compounds **1** (25 mg), **2** (30 mg), **3** (28 mg) and **4** (41 mg), respectively. All isolated compounds were for further antioxidant test except for compounds **10**-**12** due to their small amount. The structures of all the isolated compounds, except **1-4**, **6** and **7**, were elucidated on the basis of their ^1^H-NMR, ^13^C-NMR, MS, IR and UV data. Meanwhile, compounds **1-4**, **6** and **7** were identified by comparison of their *R*_f_ values on HPTLC and *R*_t_ values on HPLC with corresponding reference compounds. Moreover, the structures were proven by comparison of MS spectra of isolated and reference compounds.

### 3.6. Determination of Total Phenolic Content

The total phenolic contents were determined according to the Folin-Ciocalteu method [27] with minor modifications. Briefly, a sample of ethanol solution (0.1 mL, 1 mg/mL) was diluted with distilled water (4.5 mL) and subsequently Folin-Ciocalteu reagent (0.1 mL) was added with shaking for 3 min. A 2% (w/v) solution of sodium carbonate (0.3 mL) was added, the mixture was stirred and left to stand for 3 h. Then an aliquot of the mixture (200 μL) was transferred to 96-well plates and the absorbance was detected at 760 nm against a blank. All determinations were performed in triplicate. The total phenolic content was expressed as μg of gallic acid equivalent per mg of dry weight of the sample, using an equation obtained from the standard gallic acid calibration curve ranged from 80 to 800 μg/mL:

Absorbance = 0.0007 × gallic acid (μg) + 0.0230 (R^2^ = 0.9999)

### 3.7. Determination of Total Flavonoids Content

Total flavonoid contents were determined by the aluminum calorimetric method [28], using quercetin as the reference standard. Briefly, the test sample of ethanol solution (150 μL, 0.3 mg/mL) was mixed with 2% (w/v) AlCl_3_ (150 μL) in 96-well plates. After 15 min of incubation at room temperature, the absorbance was measured at 435 nm by spectrometer. All determinations were performed in triplicates. The content of total flavonoids was expressed as μg of quercetin equivalent per mg of dry weight of the sample, using an equation obtained from the standard quercetin calibration curve ranged from 4 to 80 μg/mL:

Absorbance = 0.0287 × quercetin (μg) − 0.0291 (R^2^ = 0.9999)

### 3.8. Determination of Antioxidant Activities

#### 3.8.1. DPPH Radical-Scavenging Activity Assay

Hydrogen-donating activity was measured by direct hydrogen donation to the DPPH radical, as previously reported, with minor modifications [29]. For each sample, different concentrations ranging from 0.6 to 500 μg/mL were prepared with methanol or 10% DMSO-methanol (v/v). The reaction mixtures in the 96-well plates consisted of sample (100 μL) and DPPH radical (100 μL, 0.2 mM) dissolved in methanol. The mixture was stirred and left to stand for 15 min in dark. Then the absorbance was measured at 517 nm against a blank. All determinations were performed in triplicates. The percentage scavenging effect was calculated as:

Scavenging rate = [1 − (A_1_ − A_2_) / A_0_] × 100%

where A_0_ is the absorbance of the control (without sample) and A_1_ is the absorbance in the presence of the sample, A_2_ is the absorbance of sample without DPPH radical. The scavenging ability of the samples was expressed as IC_50_ value, which is the effective concentration at which 50% of DPPH radicals were scavenged. The IC_50_ values were calculated from the relationship curve of scavenging activities (%) versus concentrations of respective sample.

#### 3.8.2. ABTS Radical-Scavenging Activity Assay 

The ABTS radical scavenging activity assay was carried out via the ABTS cation radical decolorization with minor modifications [30]. The samples were prepared in the same procedure as the DPPH assay. The ABTS cation radical was prepared by reacting 7 mM aqueous solution of ABTS (15 mL) with 140 mM potassium persulphate (264 μL). The mixture was allowed to stand in dark at room temperature for 16 h before use. Prior to assay, the ABTS working reagent was diluted with methanol to give an absorbance of 0.70 ± 0.02 at 734 nm and was equilibrated at room temperature. The reaction mixtures in the 96-well plates consisted of sample (50 μL) and the ABTS methanol working solution (100 μL). The mixture was stirred and left to stand for 10 min in dark, then the absorbance was taken at 734 nm against a blank. All determinations were performed in triplicate. The percentage scavenging effect was calculated as:

Scavenging rate = [1 − (A_1_ − A_2_) / A_0_] × 100%

Where A_0_ is the absorbance of the control (without sample) and A_1_ is the absorbance in the presence of the sample, A_2_ is the absorbance of sample without ABTS working solution. The scavenging ability of the samples was expressed as IC_50_ value, which is the effective concentration at which 50% of ABTS radicals were scavenged. The IC_50_ value was calculated from the scavenging activities (%) versus concentrations of respective sample curve.

#### 3.8.3. Ferric Reducing/Antioxidant Power (FRAP) Assay 

The FRAP assay was carried out according to the previously reported procedure with slight modifications [31]. Each sample was dissolved in ethanol or 10% DMSO-ethanol (v/v) to prepare the stock solution (1 mg/mL). Briefly, the working FRAP reagent was prepared by mixing 300 mM acetate buffer (pH 3.6), a solution of 10 mM 2,4,6-tripyridyl-s-triazine (TPTZ) in 40 mM hydrochloric acid and 20 mM ferric chloride at 10:1:1 (v/v/v). The working FRAP reagent (270 μL) and sample solutions (30 μL) were mixed in 96-well plates and warmed at 37 °C in constant temperature oven for 4 min. The absorbance was taken at 593 nm. Standard calibration curve was determined using different concentrations of trolox ranged from 0.093 to 3.0 μg/mL. All solutions were prepared and used on the same day. The results were corrected for dilution and calculated using the standard calibration curve (R^2^ = 0.9994) and expressed as FRAP value (μg Trolox μg-1 sample dry weight). All determinations were performed in triplicate.

## 4. Conclusions

In this study, we found that the ethanolic extract from propolis collected in Anhui, China possessed radical-scavenging and antioxidant activities. To further determine the origin of such activities, individual fractions were evaluated by three distinct assays. Our results revealed that the ethyl acetate fraction showed significant antioxidant and free radical-scavenging capacities. Furthermore, twenty-two compounds were isolated from the ethyl acetate fraction. Among them, caffeic acid (**1**), galangin (**15**), phenethyl caffeate (**16**), cinnamyl caffeate (**17**) and benzyl caffeate (**18**) showed excellent free radical scavenging activity in the DPPH assay; caffeic acid (**1**), (*E*)-*p*-coumaric acid (**2**), isoferulic acid (**3**), galangin (**15**), phenethyl caffeate (**16**), cinnamyl caffeate (**17**) and benzyl caffeate (**18**) possessed good free radical scavenging activity in the ABTS assay; caffeic acid (**1**), phenethyl caffeate (**16**), cinnamyl caffeate (**17**) and benzyl caffeate (**18**) exhibited significant ferric reducing power. Our study clearly demonstrated a significant contribution of phenolic compounds to the antioxidant activity of propolis collected in Anhui, China. Our results also confirmed that propolis collected in Anhui, China can be used as an accessible source of natural antioxidants and as a dietary nutritional supplement to promote human health and prevent oxidation-related diseases. Further investigations on the relationship between chemical constituents and antioxidant activity of Chinese propolis from different origin are underway.

## Figures and Tables

**Table 1:** Total phenolic and total flavonoids content of ethanolic extract and three fractions from propolis collected in Anhui, China. ^a^

Sample ^b^	Total phenolic content ^c^	Total flavonoids content ^d^
EE	174.7 ± 3.0	45.1 ± 0.2
CF	195.7 ± 1.5	52.4 ± 0.7
EA	235.6 ± 1.7	62.1 ± 0.7
BT	218.6 ± 0.8	34.5 ± 0.2

**^a^**: Data are mean ± standard deviation (*n* = 3). **^b^**: EE, CF, EA, BT represent the ethanolic extract, chloroform fraction, ethyl acetate fraction and *n*-butanol fraction of Anhui propolis, respectively. **^c^**: The results were expressed in μg gallic acid per mg sample dry weight. **^d^**: The results were expressed in μg quercetin per mg sample dry weight.

**Table 2:** The antioxidant activities of the selected compounds from propolis collected in Anhui, China. ^a^

Compounds	DPPH (μg/mL)	ABTS (μg/mL)	Equivalent Trolox Amount (mg/mg)
Caffeic acid (**1**)	4.91 ± 0.00	4.73 ± 0.02	3.10 ± 0.04
(*E*)-*p*-coumaric acid (**2**)	inactive	23.46 ± 0.01	inactive
Isoferulic acid (**3**)	inactive	21.62 ± 0.10	inactive
Galangin (**15**)	17.02 ± 0.02	8.73 ± 0.02	inactive
Phenethyl caffeate (**16**)	5.87 ± 0.01	10.20 ± 0.30	5.82 ± 0.08
Cinnamyl caffeate (**17**)	9.56 ± 0.01	14.60 ± 0.05	1.78 ± 0.03
Benzyl caffeate (**18**)	8.31 ± 0.01	9.91 ± 0.02	1.39 ± 0.02
Vc ^b^	5.81 ± 0.02	2.37 ± 0.00	2.23 ± 0.00
Trolox ^b^	6.84 ± 0.00	2.81 ± 0.01	−
Rutin ^b^	5.77 ± 0.03	19.43 ± 0.05	1.21 ± 0.01

**^a^**: Data are mean ± standard deviation (*n* = 3). **^b^**: Positive control.
